# Construction and evaluation of a neurofeedback system using finger tapping and near-infrared spectroscopy

**DOI:** 10.3389/fnimg.2024.1361513

**Published:** 2024-04-25

**Authors:** Shingo Takahashi, Daishi Takahashi, Yuki Kuroiwa, Noriko Sakurai, Naoki Kodama

**Affiliations:** ^1^Department of Healthcare Informatics, Faculty of Health and Welfare, Takasaki University of Health and Welfare, Takasaki, Japan; ^2^Department of Radiological Technology, Faculty of Medical Technology, Niigata University of Health and Welfare, Niigata, Japan

**Keywords:** near-infrared spectroscopy, neurofeedback, portable wearable optical topography device, finger tapping, prefrontal cortex

## Abstract

**Introduction:**

Neurofeedback using near-infrared spectroscopy (NIRS) has been used in patients with stroke and other patients, but few studies have included older people or patients with cognitive impairment.

**Methods:**

We constructed a NIRS-based neurofeedback system and used finger tapping to investigate whether neurofeedback can be implemented in older adults while finger tapping and whether brain activity improves in older adults and healthy participants. Our simple neurofeedback system was constructed using a portable wearable optical topography (WOT-HS) device. Brain activity was evaluated in 10 older and 31 healthy young individuals by measuring oxygenated hemoglobin concentration during finger tapping and neurofeedback implementation.

**Results:**

During neurofeedback, the concentration of oxygenated hemoglobin increased in the prefrontal regions in both the young and older participants.

**Discussion:**

The results of this study demonstrate the usefulness of neurofeedback using simple NIRS devices for older adults and its potential to mitigate cognitive decline.

## 1 Introduction

Neurofeedback is a form of biofeedback that allows individuals to control their brain function by measuring neural activity and presenting this information in real-time (Ehlis et al., 2018; Sitaram et al., 2019). The method is typically used to record brain waves and provide feedback (Marzbani et al., 2016), and near-infrared spectroscopy (NIRS) devices are new tools for neurofeedback training (Ehlis et al., 2018). As confirmed in healthy people, it is possible to control hemodynamic responses in prefrontal brain regions even after several training sessions of NIRS feedback (Barth et al., 2016). Neurofeedback using a small NIRS device has also been performed (Nouchi et al., 2021) and has been shown to be useful.

Neurofeedback using NIRS is free of cumbersome restrictions on participants' movement and can improve cognitive domains in patients with stroke (Renton et al., 2017). Furthermore, the method has been shown to enhance gait and balance recovery after stroke (Mihara et al., 2021). Neurofeedback approaches based on electroencephalography (EEG) and functional magnetic resonance imaging (fMRI) have been studied in older populations (Trambaiolli et al., 2021), but there are few reports on NIRS neurofeedback in this group. Neurofeedback can potentially improve cognitive function in dementia and mild cognitive impairment (Trambaiolli et al., 2021), and neurofeedback in older people using small NIRS devices can contribute to improvements in cognitive dysfunction.

Finger-tapping performance has been shown to decline among older individuals as the brain and cognitive functions deteriorate (Suzumura et al., 2021; Sugioka et al., 2022). In addition, finger tapping is effective in improving the activities of daily living (ADL) (Liu et al., 2018), and hand training may improve dexterity and executive function and, over the long term, cognitive function (Seol et al., 2023). In general, older adults have been shown to engage a broader range of brain regions for motor control than younger adults, particularly prefrontal regions and basal ganglia networks, and motor control becomes more dependent on cognition and the prefrontal cortex (PFC) with aging (Seidler et al., 2010). These findings suggest that finger tapping may be assessed within the framework of cognitive decline in older individuals.

In this study, we constructed a simple neurofeedback system using finger tapping and NIRS and examined whether brain activity improves after training. Our objectives were as follows: (1) we tested whether neurofeedback can be performed using a portable NIRS device; (2) we tested whether older participants can perform neurofeedback training during finger tapping; and (3) we tested whether prefrontal activation occurs.

## 2 Materials and methods

### 2.1 Participants and methods

In this cross-sectional study, we constructed a simple neurofeedback system and evaluated brain activity during neurofeedback implementation using a finger-tapping task. Ten older community members (four males, six females, age: 76.6 ± 5.8, 10 right-handed) and 31 healthy young adults (13 males, 18 females, age: 20.3 ± 1.3, 30 right-handed and one left-handed) were recruited to assess brain activity using the proposed system. Patients were verbally asked if they had been diagnosed with diagnosed dementia, neurodegenerative diseases, or complications, and cases, where applicable, were considered for exclusion. Patients with orthopedic, cerebrovascular, neurologic, motor, limb or finger disorders were also considered for exclusion; however, no participants were excluded. Based on the results of previous studies, a sample size of at least 15 participants was required (Takahashi et al., 2018, 2022). This sample size could not be met for the older population.

**Figure 1:**
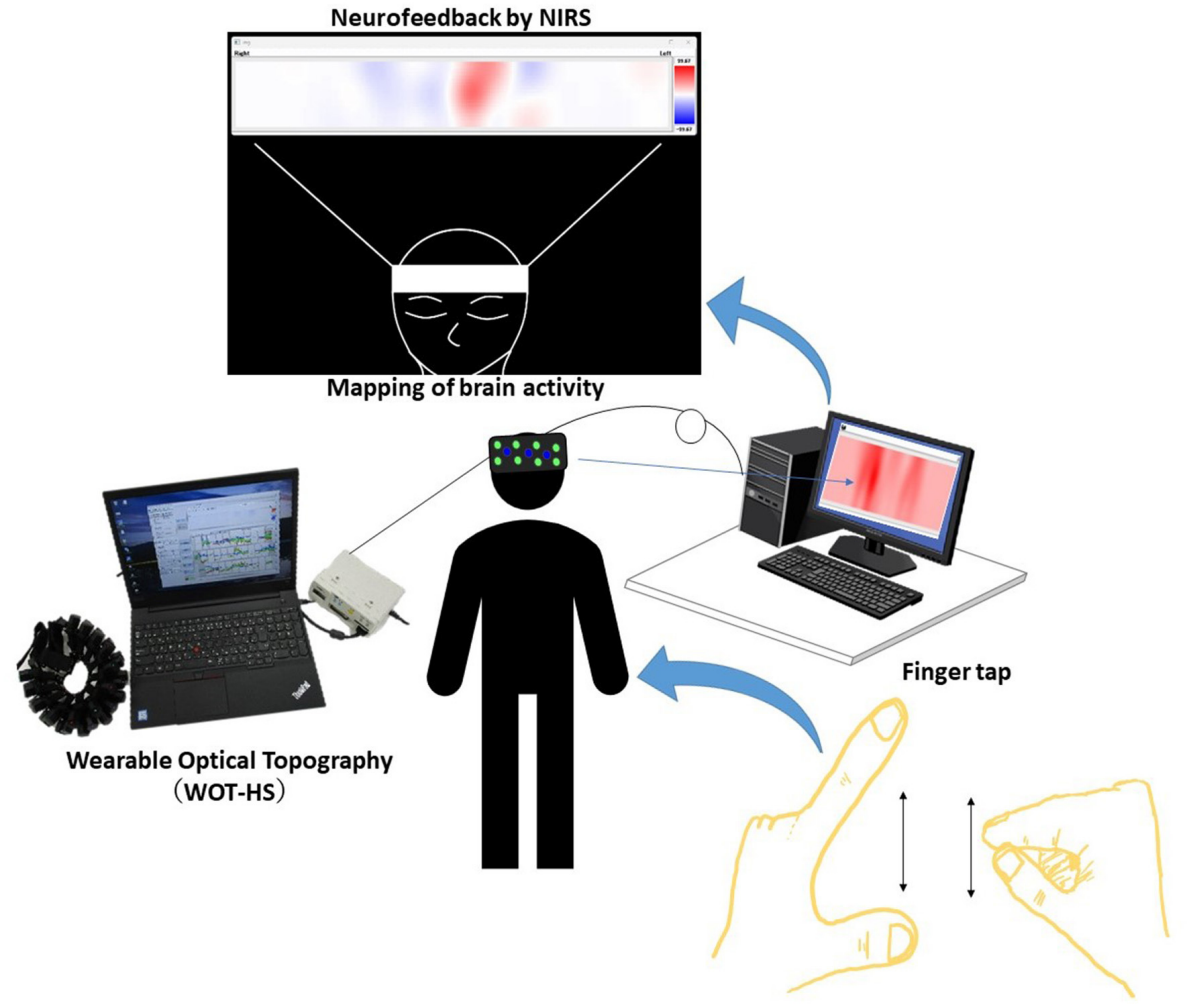
A schematic of the system. Data measured using the wearable optical topography system (WOT-HS) can be presented to the participants in real time. Changes in hemoglobin concentration (a proxy for brain activity) measured in the WOT-HS headset (34 channels) are presented on a screen. The figure at the top of the image (mapping of brain activity) is the actual screen presented to the participant. Increased and decreased hemoglobin concentrations are mapped in red and blue, respectively, while unchanged levels are shown in white.

Data from a wearable optical topography system (WOT-HS, NeU) was analyzed during neurofeedback implementation to assess brain activity during neurofeedback implementation. For neurofeedback execution and measurement, we used a three-block design with 15-s rest, 15-s tasks (neurofeedback), 15-s rest periods, and 15-s rest periods before and after the block design (Figure 1). For neurofeedback training, visual feedback based on the participant's real-time brain activity was presented on screen and finger tapping was performed with alternating hands to increase brain function (Hou et al., 2021; Nouchi et al., 2021). In the rest period, the participants were instructed to rest, with only a cross-shaped symbol appearing on the monitor during measurement.

This study was conducted in accordance with the Declaration of Helsinki. All participants were informed of the study before participation, and written informed consent was obtained. The Ethical Review Committee of Takasaki University of Health and Welfare approved the study.

### 2.2 NIRS-mediated neurofeedback system

**Figure 2:**
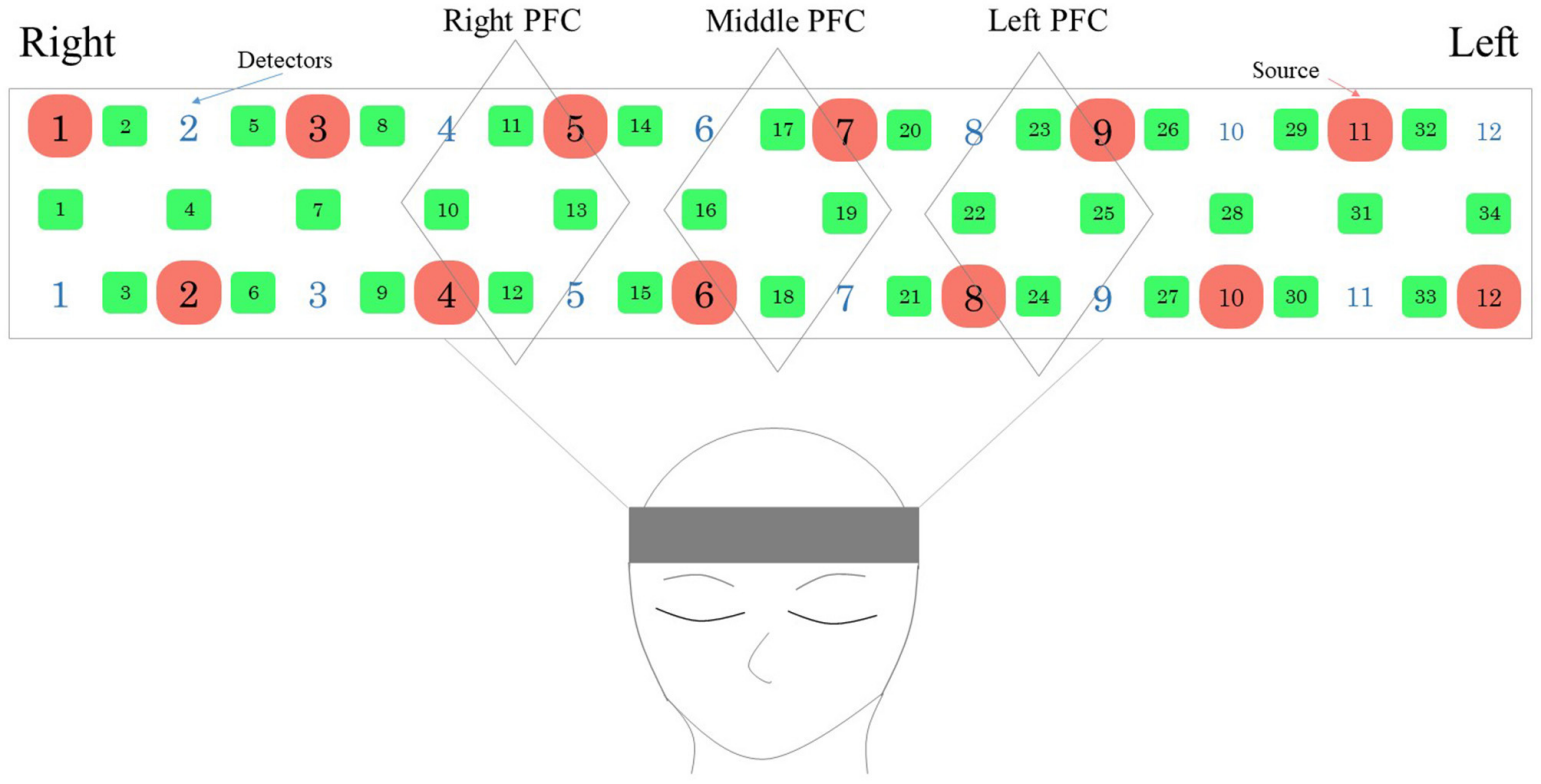
Probe placement in the WOT-HS headset. The arrangement consists of a light-emitting center and a light-receiving sensor (the sensor receives light emitted from the adjacent light-emitting center). Green numbers indicate measurement channels, red and white numbers indicate light source and detector. PFC, prefrontal cortex.

**Figure 3:**
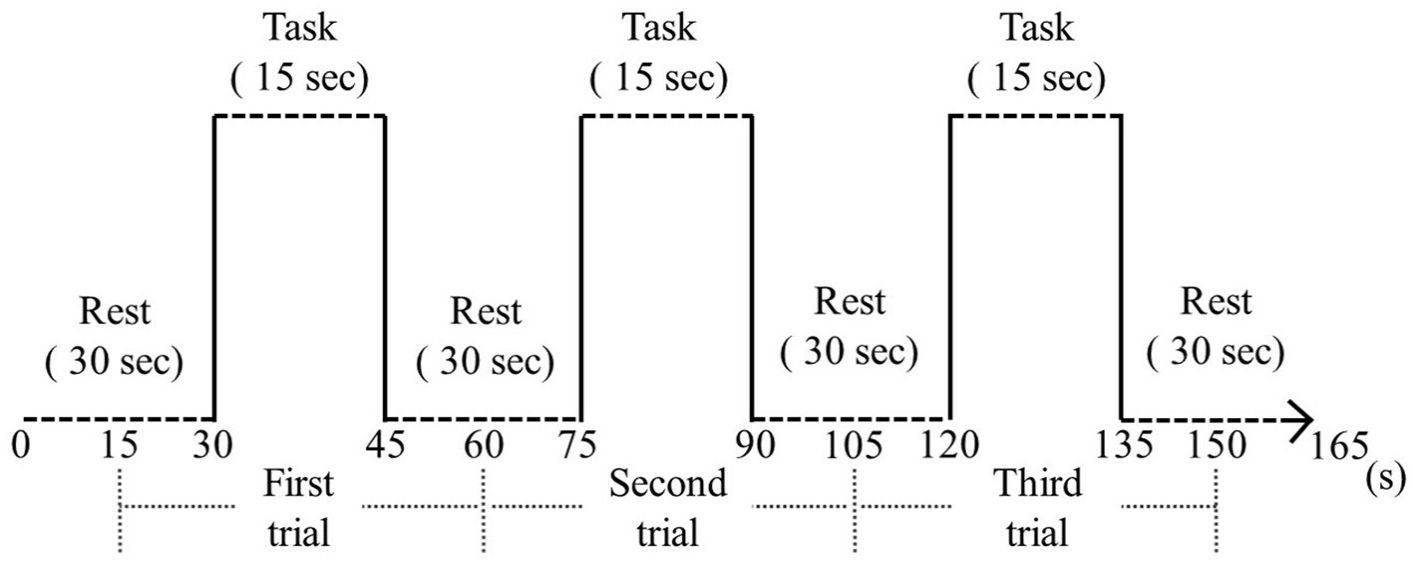
Block design. During the resting phase, a cross symbol was presented on the monitor and the participant was instructed to rest. During the task, finger tapping and neurofeedback were performed.

We constructed a simple neurofeedback system that uses a portable WOT system and presents brain activity using a programming language (Python). In detail, we created a program to present a brain activity screen using Python from the WOT-HS measurement software and constructed a simple neurofeedback method using NIRS. This system consisted of a NIRS-based measurement device, a computer for presenting brain activity, and a monitor for visualizing feedback. A schematic of the system is shown in Figure 2. NIRS captures hemoglobin signal changes derived from local vascular responses due to neuronal activation in the brain (Hoshi and Tamura, 1993; Villringer et al., 1993), and these changes were used to provide feedback on brain activity. The WOT-HS comprises a headset, a data processing unit, and measurement software. Each participant wore a headset and sat on a chair for the experiment. The headset was worn according to the instructions in the WOT-HS manual, and the participants were instructed not to move their heads during measurement. The 34 measurement channels of the WOT-HS can measure changes in oxygenated hemoglobin, deoxygenated hemoglobin, and total hemoglobin levels in the frontal and temporal regions (Figure 3). The light sources were 730 and 850 nm, and the sampling rate was 100 ms, allowing removal of signals related to skin blood flow. A dedicated application software was used for measurement, and the waveform data and mapping images were displayed in real-time. The mapped brain activity was presented to the participants as feedback.

### 2.3 Finger-tapping task

Finger tapping took the form of alternating tapping with both hands, using the thumb and index finger, with one finger closed on the left and right side and the other finger open on the left and right side (Suzumura et al., 2016; Tomita et al., 2020; Sugioka et al., 2022). The participants were instructed to place their forearms on a desk before them and tap as quickly as possible in an alternating finger opening and closing motion using their index finger and thumb for 15 s. In the open-finger configuration, they were instructed to open their fingers ~4 cm apart to reduce inter-participant variability in movement amplitude (Tomita et al., 2020).

### 2.4 Data preprocessing in NIRS and data analysis

First, the NIRS signal was bandpass filtered at 0.01–0.90 Hz from the settings at the time of measurement (Klein and Kranczioch, 2019). Next, linear fitting was performed using the values at the first and last 30 s of rest to remove drift. In this correction, a least squares method was used to estimate a linear trend from the first and last rests, and the estimated value was subtracted from the data (Xu et al., 2015; von Lühmann et al., 2020). The NIRS signal was then normalized using a z-score transformation for inter-participant comparison (Megumi et al., 2023); for the z-score, the signal at each time point was divided by the mean value during the 10 s before the first task and then by the standard deviation during the 10 s before the task. Values outside ±2 times the standard deviation of the mean were excluded as outliers (Takahashi et al., 2022). To evaluate prefrontal activation, channels (CH)10–13 were analyzed as right PFC, CH16–19 as middle PFC, and CH22–25 as left PFC. The Shapiro–Wilk test was used to verify the normal distribution of the data and perform each analysis. Since the block design was such that each participant repeated each task's conditions thrice to assess the brain activity, the data between each block were added and averaged, and the two conditions of rest and task were compared. For the comparison of rest and tasks in older adults and young participants, the Wilcoxon rank-sum test was performed. We used the SPSS software (version 27.0 for Windows; IBM Corp., Armonk, NY, USA) for the statistical analyses. For multiple comparisons, the Bonferroni correction set the significance level at < 0.0167%.

## 3 Results

**Figure 4:**
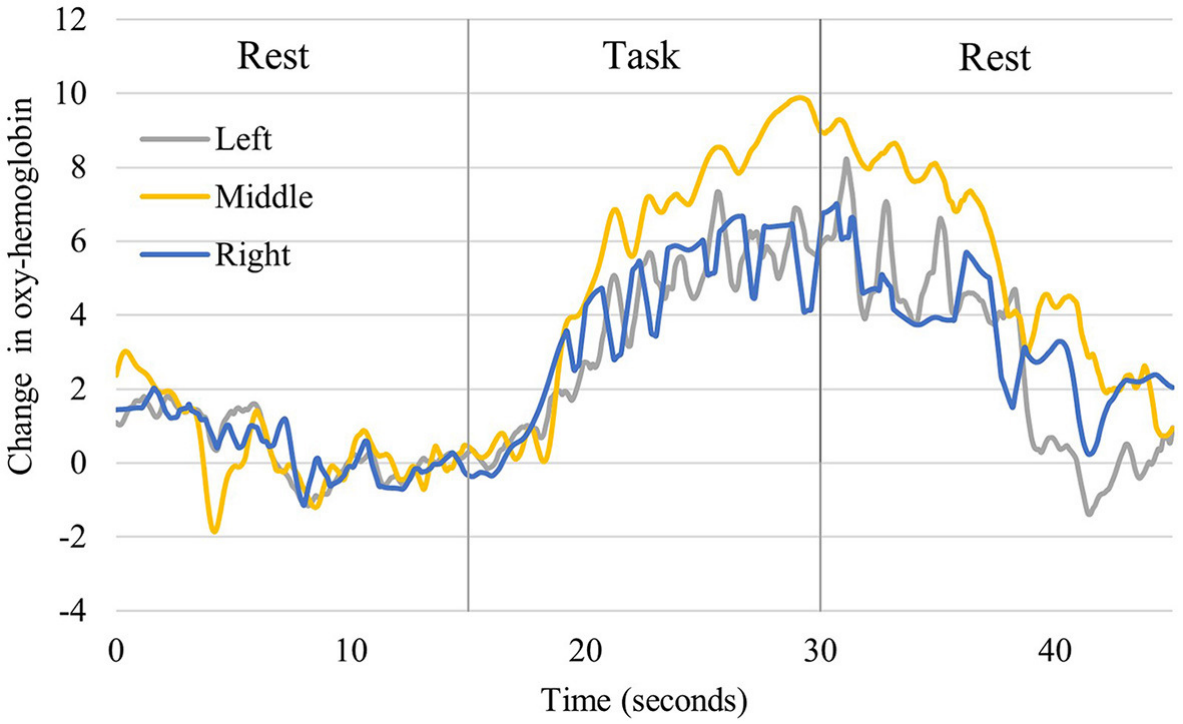
Changes in oxyhemoglobin levels recorded from right, middle, and left channels in older participants. It shows one representative person who showed significant activation. The blue line shows the values for the right, the yellow line for the central, and the gray line for the left prefrontal region.

Figure 4 shows the time course of changes in oxygenated hemoglobin levels in the older participants (One typical example of significant brain activation). The signal tended to increase during the implementation of the task (neurofeedback), as observed in the younger participants. At rest, after the implementation of the task, the oxygenated hemoglobin levels decreased. During the task implementation, there was an increasing trend in all left, central, and right PFC regions. Table 1 presents a comparison of the mean values in the older adults at rest before the task and during the task. The rest of the Right PFC in older adults had a mean of 0.1078 with a standard deviation of 0.2831, and the task had a mean of 1.0486 with a standard deviation of 1.1158. The z-value of the test was 2.497, and the *p*-value was 0.013, indicating a significant difference between rest and task. In Middle PFC, the rest had a mean of 0.1209 with a standard deviation of 0.1777, and the task had a mean of 0.7175 with a standard deviation of 1.7076. The test resulted in a z-value of 1.274 and a *p*-value of 0.203, indicating no significant difference between rest and task. In the Left PFC, the rest had a mean of 0.0247 and a standard deviation of 0.2904, while the task had a mean of 0.4243 and 1.3741. The test resulted in a z-value of 1.172 and a *p*-value of 0.241, indicating no significant difference between rest and task.

**Table 1:** Oxyhemoglobin concentration values during each task period in older participants.

	**Rest**		**Task (neurofeedback and finger tap)**		***z*-value**	***p-*value**
	**Average (95% CI)**	**SD**	**Average**	**SD**		
Right PFC	0.1078 (−0.0606-0.2691)	0.2831	1.0486 (0.5102-1.7592)	1.1158	2.497	0.013^*^
Middle PFC	0.1209 (0.0135-0.2235)	0.1777	0.7175 (0.0665-1.8358)	1.7076	1.274	0.203
Left PFC	0.0247 (−0.1668-0.1814)	0.2904	0.4243 (−0.2648-1.2998)	1.3741	1.172	0.241

^*^p < 0.0167%. CI, confidence interval; PFC, prefrontal cortex; SD, standard deviation.

**Figure 5:**
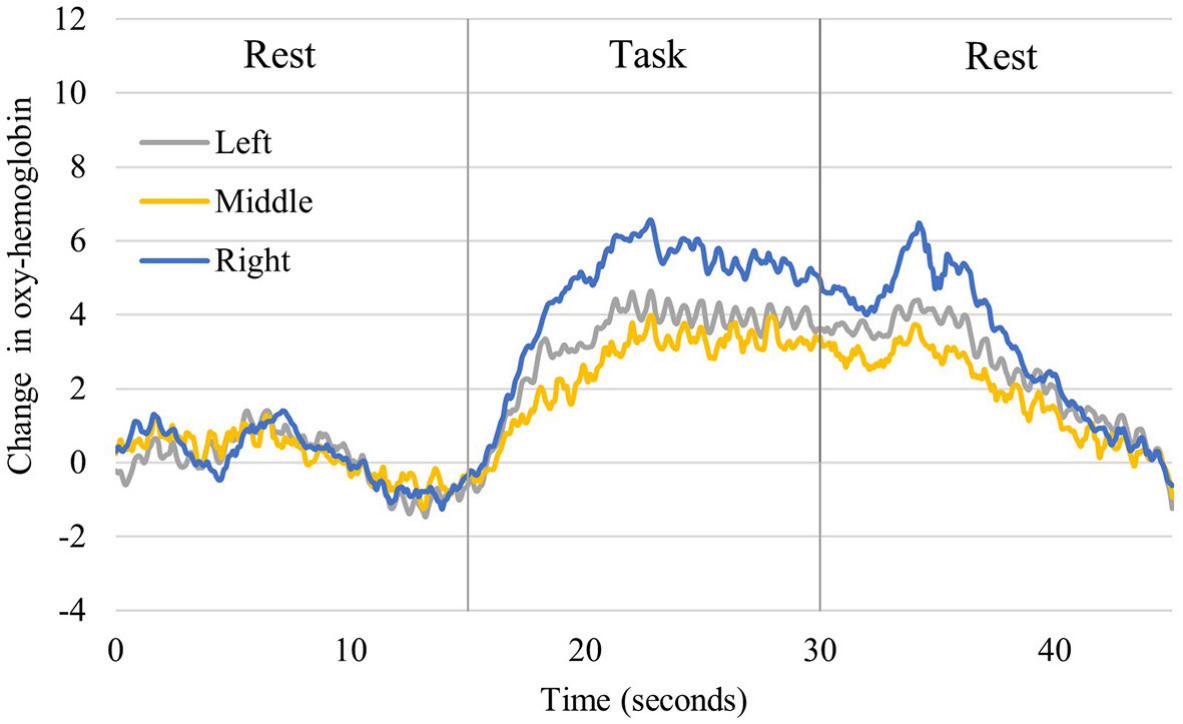
Changes in oxyhemoglobin levels recorded from right, middle, and left channels in young participants. It shows one representative person who showed significant activation. The blue line shows the values for the right, the yellow line for the central, and the gray line for the left prefrontal region.

Figure 5 shows the time course of the changes in oxygenated hemoglobin levels in healthy young participants (one typical example of significant brain activation). Oxidized hemoglobin levels tended to increase from before the task to when neurofeedback was implemented, followed by a decrease from neurofeedback implementation to the resting state. All left, central, and right prefrontal regions showed an increasing trend during neurofeedback implementation. Table 2 compares the mean values at rest before the task and during neurofeedback. The rest of the Right PFC for young adults had a mean of 0.0004 with a standard deviation of 0.002, and the task (neurofeedback and finger tap) had a mean of 2.4799 with a standard deviation of 4.3473. In Middle PFC, Rest had a mean of 0.0033 and a standard deviation of 0.0183, while the task had a mean of 0.7957 and a standard deviation of 6.446. The test resulted in a *t-*value of −0.685 and a *p*-value of 0.498, indicating no significant difference between rest and task. In the Left PFC, the rest had a mean of −0.0023 and a standard deviation of 0.0151, while the task had a mean of 1.2267 and a standard deviation of 4.2893. The test resulted in a *t*-value of −1.595 and a *p*-value of 0.121, indicating no significant difference between rest and task. From Tables 1, 2, both older and young adults, there were no significant differences between the pre-task resting period and the neurofeedback period in the left and central prefrontal regions, while significant differences in the right prefrontal region were demonstrated.

**Table 2:** Oxyhemoglobin concentration values during each task in young.

	**Rest**		**Task (neurofeedback and finger tap)**		***z*-value**	***p-*value**
	**Average (95% CI)**	**SD**	**Average**	**SD**		
Right PFC	0.0004 (−0.0003-0.0012)	0.0020	1.9770 (0.775-3.1789)	3.2769	4.056	< 0.001^*^
Middle PFC	0.0033 (−0.0034-0.01)	0.0183	0.4454 (−1.5363-2.4272)	5.4028	1.431	0.153
Left PFC	−0.0023 (−0.0078-0.0032)	0.0151	0.9188 (−0.2394-2.077)	3.1574	1.352	0.176

^*^p < 0.0167%. CI, confidence interval; PFC, prefrontal cortex; SD, standard deviation.

## 4 Discussion

In this study, we developed a simple NIRS-based neurofeedback system and evaluated brain activity during its implementation. Neurofeedback using NIRS can either increase or decrease brain activity (Hosseini et al., 2016; Kinoshita et al., 2016; Kohl et al., 2022), and this system can respond to both types of feedback. The presentation screen can also show brain activity using mapping, waveforms, or bars. In our system, brain activity was indicated using mapping. The measurement screen can also display waveforms, allowing visualization of both maps and waveforms.

In a previous study that developed an NIRS neurofeedback system, activation in cerebral regions, including PFC, was assessed during real-time neurofeedback (Kinoshita et al., 2016). In the previous study, using a smaller device, we also found that neurofeedback altered brain activity bilaterally in dorsolateral (DL)PFC during cognitive training (Nouchi et al., 2021). In our study, we observed a significant difference between rest and task in the right PFC, as well as increasing trends in other areas. However, a degree of variation was noted. We found an increase in oxygenated hemoglobin concentration during neurofeedback, suggesting that our results are similar to those of previous studies. Furthermore, the present results suggest that neurofeedback can be effective, even with this simple system. Our results showed a significant difference only in the right PFC. This may be because the present method involved visuospatial cognitive abilities, which are related to the ability to grasp and visualize own brain activity based on brain activity mapping images. Since visuospatial processing ability is dominant in the right hemisphere (Kwon et al., 2002; Corballis, 2003; Suzuki et al., 2018), a greater activation was observed in the right PFC than in other regions, which may have resulted in a significant difference. Other than this, we could not show any other influence directly related to the significant difference in the right PFC.

Circuits involving DLPFC, PFC, and the cerebellum have been reported to control motor accuracy (Torriero et al., 2007; Abiru et al., 2016). Since finger tapping in the present study involved alternating movements, prefrontal regions were likely involved. However, the areas that control finger movement traditionally include the primary motor cortex (BA4), premotor cortex, supplementary motor area (BA6) (Sugioka et al., 2022), primary sensorimotor cortex, and the cerebellum (Turesky et al., 2018). Activation in prefrontal regions during alternating finger tapping has not been confirmed in young individuals (Takahashi et al., 2022). In our paradigm of finger tapping with neurofeedback, the concentration of oxygenated hemoglobin increased, which may also be due to the effect of this system. We believe the proposed method, which seeks to increase brain activity while performing alternating finger tapping, has a double-task element. Dual tasks that involve exercise and cognitive paradigms, such as walking, activate PFC (Holtzer et al., 2011; Kvist et al., 2023), and even in older people with mild cognitive impairment, PFC activation has been observed during dual-task walking (Doi et al., 2013). Since the method in this study may comprise a dual-task element, brain activity is expected to increase during neurofeedback.

Neurofeedback with NIRS has been shown to have strong effects, avoiding non-trivial restrictions on participants' movements, and neurofeedback therapy in patients with stroke has been shown to improve cognitive domains in these patients (Renton et al., 2017). DLPFC activation can enhance the benefits of cognitive training (Nouchi et al., 2020); in other words, it is important to increase DLPFC activity during cognitive training to enhance cognitive function (Nouchi et al., 2021). The results of this study show that neurofeedback during finger tapping enhances brain activity even in older adults. Therefore, there is potential for the prevention of dementia, and future studies are needed for more in-depth evaluations.

This study had three notable limitations. The first issue is the number of older participants. Comparisons should be made between groups of younger and older adults, as well as between older adults and cognitively impaired participants; it will be necessary to increase the number of participants and conduct additional surveys in the future. Second, this was a cross-sectional study. Previous studies on neurofeedback have evaluated function before and after intervention and reported improvements in motor function. Although other cross-sectional studies have been performed, it is desirable to conduct longitudinal measurements to evaluate improvements in brain, cognitive, and motor function. Third, we used a specific task and measurement site: we focused only on the finger-tapping task and on recording changes in prefrontal regions, but neurofeedback may be effective using other tasks and measurements in other brain regions. It is also necessary to measure task performance to further verify the effectiveness of neurofeedback.

In conclusion, a simple neurofeedback system using finger taps and NIRS was constructed to measure oxygenated hemoglobin concentration during finger taps and neurofeedback in older participants and young. In this system, the concentration of oxygenated hemoglobin during neurofeedback implementation in the two groups of participants increased. Further studies are needed to investigate the usefulness of this system in older populations.

## Data availability statement

The raw data supporting the conclusions of this article will be made available by the authors, without undue reservation.

## Ethics statement

The studies involving humans were approved by the Ethical Review Committee of Takasaki University of Health and Welfare. The studies were conducted in accordance with the local legislation and institutional requirements. The participants provided their written informed consent to participate in this study.

## Author contributions

ST: Writing – review & editing, Writing – original draft, Methodology, Investigation, Funding acquisition, Formal analysis, Data curation, Conceptualization. DT: Writing – review & editing, Writing – original draft, Investigation, Data curation. YK: Writing – review & editing, Writing – original draft, Methodology, Investigation. NS: Writing – review & editing, Writing – original draft, Formal analysis, Conceptualization. NK: Writing – review & editing, Writing – original draft, Validation, Conceptualization.
